# Coupled Au Nanoparticle-Cavity Nanostructures for Precise Control in Resonance-Driven Photocatalytic Reactions

**DOI:** 10.1021/acsnano.5c04020

**Published:** 2025-07-11

**Authors:** Ning Lyu, Anjalie Edirisooriya, Zelio Fusco, Shenyou Zhao, Fiona J. Beck, Christin David

**Affiliations:** † Institute of Solid State Theory and Optics, 9378 Friedrich-Schiller-Universität Jena, 07743 Jena, Germany; ‡ School of Engineering, 2219 Australian National University, Acton ACT 2601, Australia; § School of Electronic Engineering, Xi’an University of Posts and Telecommunications, Xi’an 710121, China; ∥ University of Applied Sciences Landshut, Am Lurzenhof 1, 84036 Landshut, Germany

**Keywords:** localized surface plasmonic resonance (LSPR), Fabry−Pérot nanocavity resonance, strong coupling regime, self-assembly nanoparticles, photocatalysis, resonance-driven reactions

## Abstract

Photocatalysis offers a sustainable approach to converting solar energy into chemical energy, enabling the production of renewable fuels and chemicals with net-zero emissions, a crucial step toward a renewable energy-based economy. Recent advancements in nanophotonics, particularly in plasmonic hybridized nanostructures, have enabled tunable localized surface plasmon resonances, offering solutions for selective, resonance-driven chemical applications via two nonthermal mechanisms: near-field enhancement, which amplifies the localized electromagnetic field, and hot electron energy transfer, which injects energetic electrons into reactants. We designed a series of self-assembled Au nanoparticle cavities to precisely control plasmonic resonance strength via Fabry–Pérot (F–P) resonances by tuning the TiO_2_ cavity thickness. The strong coupling between plasmonic and F–P modes can be strategically exploited to either enhance or suppress a model reaction, the photodegradation of methylene blue. By tuning the F–P node or peak to achieve spatial and spectral overlap with the plasmonic resonance, we can facilitate and enhance the reaction. Specifically, this approach enhances the product yield by a factor of 102, from 0.07 to over 7.18, as determined by the integration of the vibrational peak of the product at 480 cm^–1^ in the Raman spectrum. These findings demonstrate that plasmonic hybridized nanostructures enable control over reactions to modulate the desired product yield. In this work, we demonstrate a strategy for optically manipulating reaction rates to either enhance target products or suppress it. This approach advances the selective control of photocatalysis, offering opportunities to enhance conversion processes, and has potential applications in renewable fuel production.

## Graphical abstract

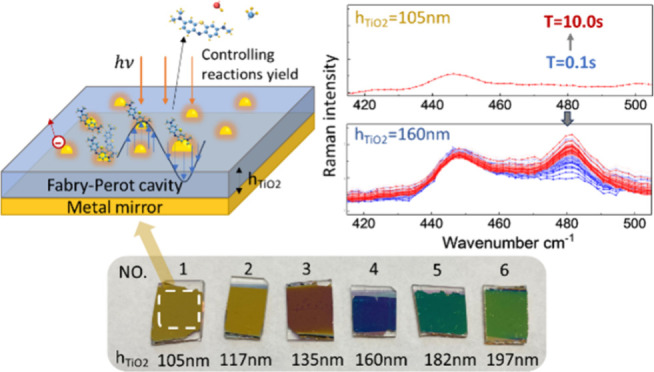

Light-driven catalytic reactions use solar energy to produce renewable fuels and chemicals with net-zero emissions.^1^ This approach holds significant potential for transitioning to a global economy powered by renewable energy, addressing both current and future energy demands across various sectors, particularly in hard-to-abate industries like mining and shipping.^2–4^ Recent advances in nanophotonics have been integrated into photochemical applications to demonstrate optical resonance-driven chemistry, particularly through plasmonic^5,6^ and hybridized nanostructure designs.^7,8^ In resonance-driven photocatalytic reactions, subwavelength nanoresonators interact with visible light to efficiently harvest solar energy for catalysis. Their tunable optical properties offer opportunities for manipulating optical energy transfer at the nanoscale.^9^

Plasmonic nanostructures are widely applied in optical resonance-driven photocatalysis due to their ability to concentrate light into small volumes via localized surface plasmon resonances (LSPR) and their intrinsic catalytic activity.^1^ In this process, electromagnetic waves at specific wavelengths excite collective oscillations of electrons within metallic nanoparticles, creating regions of strong field enhancement, referred to as ″hot spots″ on the metal surface. The oscillation of free electrons enhances the near-field around the nanoparticles, illustrated in Figure 1a, transferring energy via photons to the surrounding environment, which can include adsorbed reactant molecules. Additionally, nonradiative decay of the plasmonic oscillation excites some electrons (or holes) to energy levels above the Fermi level within the nanoparticles. During their short, picosecond lifetime, these ″hot carriers″ can be injected over an energy barrier into acceptor electronic states of nearby molecular adsorbates.^10^ If they are not transferred, hot carriers relax via electron–electron scattering, leading to local heating which can also increase reaction rates.^11^ During this process, two nonthermal charge-transfer mechanisms can occur to drive photoreactions: photon absorption in the near-field can excite electrons from the HOMO to the LUMO within the molecule initiating or enhancing chemical reactions (Figure 1a); or hot electrons can be directly (dotted line in Figure 1a) or indirectly (solid line Figure 1a) injected into molecular orbitals, activating chemical transformations.^12^ These nonthermal mechanisms of plasmonic photocatalysis—near-field enhancement and hot carrier transfer—could enable precise activation of particular chemical bonds for catalytic reaction processes.^13^

**Figure 1:**
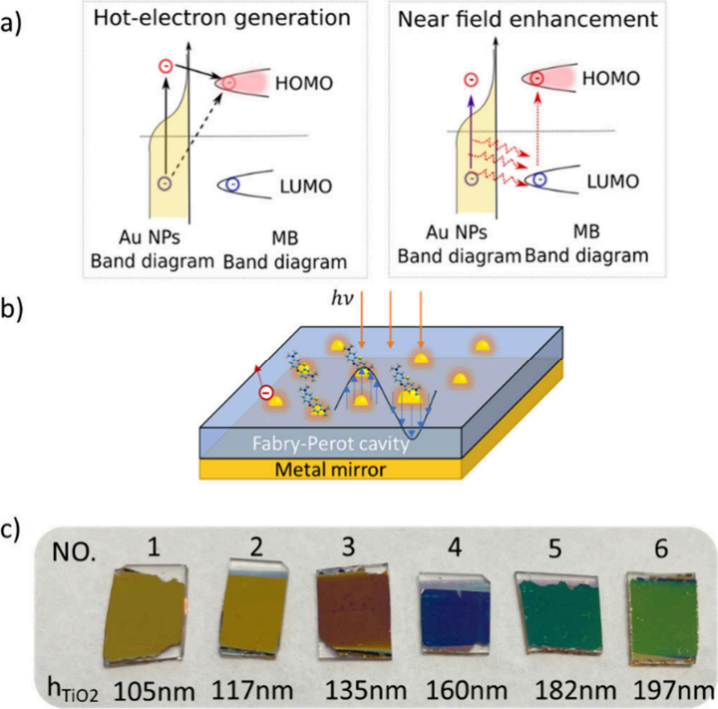
a) Mechanisms for plasmon-driven photocatalysis: hot-electron generation (left) and near-field enhancement (right) b) Configuration of Au NP-cavity. c) Au NP-cavity samples with different cavity thicknesses chosen to represent different points in the simulation discussed below. Thickness of TiO_2_ cavities were measured with an ellipsometer.

Recently, plasmonic nanostructures have been employed to enhance the conversion efficiency of photocatalytic reactions enabling wideband solar energy harvesting,^14–16^ wide-angle light absorption,^17^ efficient electron excitation,^7,8,18^ and wavelength tuning for specific reactions.^19^ Optical tunability was also employed to target specific reaction products. For example, Shi et al. designed Au nanoparticles/TiO_2_/Au-film structures to enhance plasmon-induced water-splitting reaction. With appropriate thicknesses of the TiO_2_ cavity, the Au nanoparticles (Au NPs) can meet strong coupling conditions, resulting in dual peaks in the absorption spectrum. This effect broadens the resonance wavelength range and effectively enhances the hydrogen (H_2_) evolution.^8^ Further studies have explored the ability of metasurfaces and nanostructures to achieve selectivity in photochemical processes by tailoring their optical properties. For instance, theoretical work by Wang et al. provided valuable insights into designing polarization-sensitive systems using chiral plasmonic nanostructures.^9^ These nanostructures exhibit significant chiral asymmetry in optical absorption and hot electron generation between left- and right-circular polarizations, demonstrating their potential for driving photochemical reactions.

These studies demonstrate that plasmonic nanostructures have been employed to drive photocatalytic reactions via plasmonic resonance. Their optical properties were tuned to activate and enhance specific photochemical reactions. To advance the selective control of photocatalytic reactions, it is essential for plasmonic nanostructures to enable both enhancement and suppression of the reactivity. However, a systematic investigation into the relationship between optical properties and catalytic performance remains lacking. Such a study is crucial for achieving precise selectivity in photochemical processes.

Selectivity is crucial in multibranched reactions, such as the carbon dioxide reduction reaction (CO2RR), which involves multiple steps with various intermediates branching into different pathways.^20^ Enhancing or suppressing specific intermediate steps can promote desired pathways and prevent side reactions, ensuring that target products can be produced more efficiently, enabling large-scale renewable fuel production.^21^ In optical resonance-driven reactions, near-field enhancement and hot carrier transfer can be tailored through morphological engineering of nanostructures to selectively match the energy of specific chemical bonds or unoccupied adsorbate states and manipulate the reaction conversion rate.^1,22^ However, recent studies indicate that the catalysts involved can undergo continuous structural reconstruction under practical operating conditions, leading to conflicting interpretations of the active sites and reaction mechanisms in CO_2_ reduction.^23^ Current analytical techniques face challenges in effectively monitoring the CO2RR intermediates in real-time. To demonstrate the selectivity strategy of resonance-driven reactions, the N-demethylation of methylene blue (MB) on plasmonic nanoparticles can be employed as a model reaction, which has been shown to be driven by plasmon assisted photocatalysis.^13^ Product generation rates can be monitored in real time by using surface-enhanced Raman spectroscopy (SERS).

Aiming to manipulate resonance-driven reactions, we designed a series of coupled Au nanoparticle-cavity (Au NP-cavity) nanostructures with tunable optical properties. Figure 1b–c illustrate the configuration of Au NP-cavities with reactant molecules adsorbed to the surface of plasmonic nanoparticles. The gold nanoparticles catalyze light-driven reactions within a specific wavelength range around the LSPR at approximately 659 nm in wavelength as shown in Figure S1a of the Supporting Information (SI). A semiopen optical cavity is formed due to the high reflectance of the metallic mirror film, supporting Fabry–Pérot (F–P) resonances within the anatase TiO_2_ cavity layer. These resonances can be prescriptively tuned to a particular wavelength by adjusting the cavity thickness, as shown in Figure S1b of the SI, enabling the formation of tunable constructive peaks and destructive nodes. A strong coupling regime is established in the Au NP-cavity through the interaction between the LSPR and the F–P mode, which will be discussed in the following section.

We experimentally investigate the potential of Au NP-cavities to manipulate a model plasmon-mediated photoreaction: the N-demethylation of MB, which we have previously demonstrated is driven by both near-field effects and hot-electron transfer.^13^ The hybridized resonance of the system is tuned by controlling the F–P resonance peak wavelength via the cavity thickness. **Thus, we demonstrate precise control of the product conversion rate of the MB reaction by adjusting the strength of the LSPR resonance through tuning the F–P resonance peak in the strong coupling regime.**

## Results and Discussion

### Tunable Optical Properties of Au NP-Cavity Designs

We have previously shown that the interaction between FP and LSPR modes can be well described using a simple coupled oscillator model.^24^ When the LSPR peak spectrally and spatially overlaps with the F–P resonance strong coupling occurs and the hybrid mode is subject to energy splitting, resulting in high and low energy peaks in the absorption spectrum.^8,25^ The upper, ω_*c*+_, and lower, ω_*c*–_, resonance frequency of the hybridized mode are determined by the resonance frequencies of the two coupled modes, calculated as

$$\omega_{c\pm }=\frac{\omega_{LSPR}}{2}+\frac{\omega_{mFP}}{2}\pm \frac{1}{2}\sqrt{\left(\omega_{LSPR}^{2}-\omega_{mFP}^{2}\right)+\Omega^{2}}$$

where ω*_LSPR_* and ω_*mFP*_ are the resonance frequencies of the Au NP LSPR and m-th order F–P mode of the TiO_2_ cavity, respectively. The coupling strength, Ω, represents the frequency splitting that occurs when the resonance frequencies of the two coupled modes are identical and is dependent on the nanoparticle density in coupled Au NP-cavity systems, with more dense coverage leading to stronger coupling.^24^

To demonstrate the tunability of Au NP-cavities, total absorption spectra as a function of TiO_2_ cavity thickness were calculated using the finite element method (FEM), building on the previous work of Zhao et al.^24^ Figure 2a illustrates the variations in optical absorption of the hybridized system as the TiO_2_ cavity thickness increases, while the Au NP parameters are kept constant (r = 14 nm and gap = 22 nm). Details of simulation models are given in the Method section and SI Figures S1–S4. Due to absorption in the Au mirror layer and the bandgap of anatase TiO_2_ (E_g_TiO2_ = 3.2 eV), the absorption of the nanocavity without Au NPs cuts off at approximately 510 nm (see the SI and Figure S1b for details). After adding Au NPs to the surface of the cavity, the total absorption extends to the 600–700 nm range, due to the LSPR resonance at 659 nm, which enhances the absorption in this spectral region.

**Figure 2:**
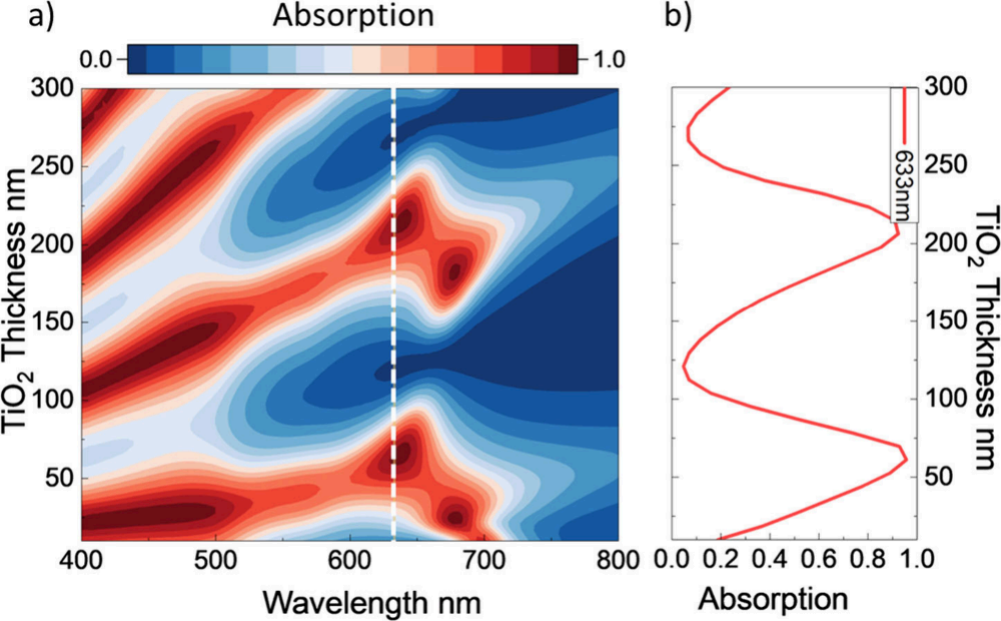
a) Simulated absorption of plasmonically enhanced nanoparticle-cavity as a function of cavity thickness and b) absorption tuning at target wavelength 633 nm.

At the target wavelength of 633 nm, corresponding to the molecular resonance (HOMO–LUMO gap) of MB, the strength of the absorption due to the hybridized resonance oscillates with an increase in the cavity thickness and hence the cavity resonance wavelength, as shown in Figure 2b. We leverage this precise control of the resonances to manipulate resonance-driven reactions. We further calculate the average near field enhancement and hot electron generation rate to evaluate and compare the two nonthermal effects as described in the SI Figure S5.^24,26^

Guided by these simulations, we fabricate six samples to explore hybridization of LSPR with the second cavity mode (m = 2), with varying TiO_2_ thicknesses from 105 to 197 nm. Figure 1c visually demonstrates spectral tunability across the samples, with their reflective color shifting from gold to green.

The cavities were fabricated by depositing anatase TiO_2_ on glass substrates that had been coated in thin gold layers to form a mirrored surface, following the process outlined in the Method section and Figure S6. The Au NPs were prepared using a self-assembly approach by evaporating and annealing thin layers of Au.^27^ The size of the Au NPs is dependent on the initial Au film thickness, which was set at a constant value of 5 nm for all of the samples. The scanning electron microscope (SEM) image inset in Figure 3a shows that the Au NPs take on a hemispherical shape with a disordered distribution and a variation in size.^28^

**Figure 3:**
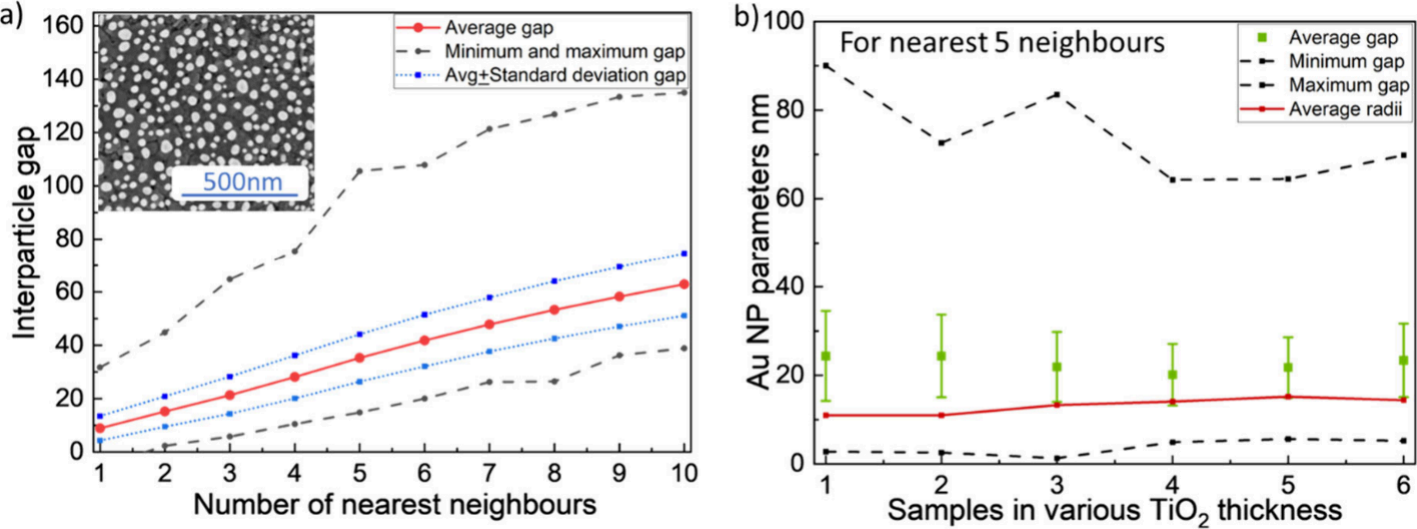
a) Interparticle gap of up to ten nearest neighbor particles of sample 5 (TiO_2_ thickness is 182 nm) showing averaged values (red solid curve), averaged values ± standard deviation (blue dotted curves), and minimum and maximum values (black dashed curves). The inset shows an SEM image of the Au NPs. b) Averaged interparticle gaps (green squares) of five of the nearest neighbor NPs in the six samples. The error bar shows the standard deviation values. Furthermore, maximum and minimum gap size (black dash curves) and averaged radii (red solid curve) of the samples are given.

Our previous work showed that disordered arrays of Au NPs could be well represented by optical simulations of periodic arrays of identical Au NPs, but that the plasmonic resonance was sensitively dependent on the radius and interparticle gap (as shown in Figures S7 and S8), leading to broader resonances for disordered arrays with some size dispersion.^24^ To estimate the size parameters for the experimental arrays, the equivalent radius of each nanoparticle was calculated based on circles with the same area as the NP in the SEM image. The histograms are in Figures S6 and S7 showing the parameter distribution for the radius and interparticle gap of the six samples, and this information is summarized in Figure 3. The average radius remains approximately stable at 14 nm across all the samples, as shown in Figure 3b. The red curve in Figure 3a represents the average interparticle gap for up to ten nearest neighboring nanoparticles in sample 5, which gradually increases from 8 to 50 nm with the number of nearest neighbors considered. Since interparticle coupling is significantly affected by distance, the average interparticle gap of the five nearest surrounding neighbors (22 nm) is used for future optical analysis. Due to the consistent fabrication conditions, the differences in geometric parameters of NPs are negligible across all samples, which are thus expected to support plasmonic resonances at similar wavelengths.

As demonstrated above, the thickness of the TiO_2_ cavity is a key parameter for varying the F–P resonance wavelength, which is precisely controlled by adjusting the sputtering duration. The relationship between the sputtering duration and cavity thickness is shown in Table S1. These thicknesses are the average values of three measurements on each sample with uncertainties. Six samples were prepared with cavity thicknesses ranging from approximately h_TiO2_ = 105 nm to h_TiO2_ = 197 nm, covering both the constructive peak and the destructive valley of F–P resonances, based on simulations shown in Figure 2.

We compare the experimentally measured (solid line) and calculated (dashed line) absorption spectra for each sample in Figure 4. Simulated NPs have parameters of r = 14 nm and gap = 22 nm, taken from the SEM data presented in Figure 3b. Absorption of the nanostructures is calculated from measured reflection and transmission spectra collected with a microspectrophotometer. In general, the simulated data well represent the trends in the experimental spectra. As an example, the design with an h_TiO2_ = 105 nm thick TiO_2_ film in Figure 4a shows suppressed absorption of 4%, at the target wavelength of 633 nm, indicated by a red vertical line. This suppression arises from the inhibition of the hybrid Au NP–cavity resonance, as the LSPR, calculated to be at 659 nm, aligns with the destructive interference valley of the F–P cavity. A similar phenomenon can be observed in the simulated spectrum with suppressed absorption of ∼4% at 633 nm. The absorption peak at wavelengths <450 nm is attributed to parasitic absorption in both the Au (mirror) and TiO_2_ film. As the cavity thickness increases in Figure 4b to f, the F–P resonance peak red-shifts, gradually enhancing the spectral and special overlap of the LSPR and F–P resonance and, hence, increasing absorption due to the hybridized mode at the target wavelength.

**Figure 4:**
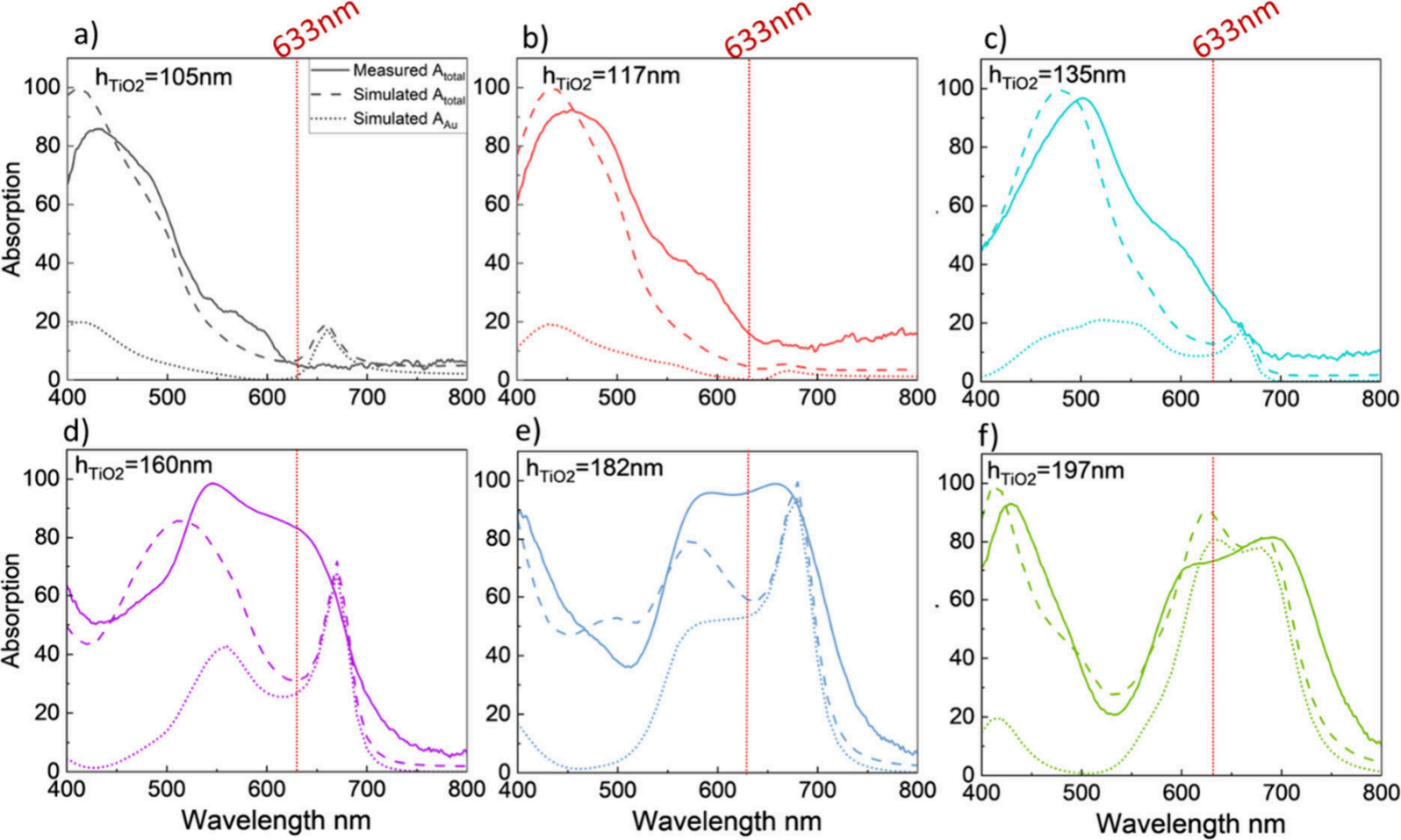
Measured (solid curves) and simulated (dashed curves) absorption of six samples with TiO_2_ thickness of a) h_TiO2_ = 105 nm, b) h_TiO2_ = 117 nm, c) h_TiO2_ = 135 nm, d) h_TiO2_ = 160 nm, e) h_TiO2_ = 182 nm and f) h_TiO2_ = 197 nm. In addition, simulated absorption in solely the Au NPs is shown as dotted curves.

When the cavity thickness reaches h_TiO2_ = 160 nm or more, in Figure 4d to f, the F–P resonance overlaps with the LSPR leading to strong coupling and resulting in energy splitting that manifests as dual absorption peaks. For the measured spectrum for the h_TiO2_ = 160 nm (Figure 4d) sample, the target wavelength aligns with the high energy peak of the hybridized mode, while for the sample with h_TiO2_ = 197 nm (Figure 4f) it aligns with the lower energy peak. For the h_TiO2_ = 182 nm sample (Figure 4e), the target wavelength falls between the F–P and LSPR peaks. Consequently, absorption at the target wavelength reaches a peak value of 92%, with a slight reduction in the h_TiO2_ = 197 nm sample.

The coupling strength, Ω, calculated as the absolute difference between two resonance frequencies (|ω_+_ + ω_–_|), characterizes the coupling efficiency of the plasmonic resonance and F–P resonance (as shown in Table S2). This coupling strength reaches a maximum of 79 GHz for the h_TiO2_ = 160 nm sample. At this thickness, the plasmonic resonance exhibits both strong spatial and spectral overlap with the cavity mode, facilitating the most efficient interaction. As the TiO_2_ thickness increases, the cavity resonance red-shifts, resulting in spectral misalignment with the plasmonic resonance. Consequently, the coupling strength reduces to 57 and 51 GHz, for h_TiO2_ = 182 nm and h_TiO2_ = 197 nm, respectively.

We also plot the simulated absorption in the Au NPs (dotted lines) as an indicator of how the hybridized resonances will drive photocatalysis of MB molecules adsorbed on the nanoparticles. The same trends are seen in Au NP absorption as in total absorption. Critically, absorption in the NP is suppressed to near zero at the target wavelength for samples with h_TiO2_ = 105 nm and h_TiO2_ = 117 nm and reaches a maximum of 81% for the sample with h_TiO2_ = 197 nm. For the h_TiO2_ = 160 nm sample, the lower-energy peak in the simulated spectrum exhibits a slight red-shift of approximately 10 nm compared to the experimentally measured total absorption. This is likely due to the limitation that the simulated arrays are represented by a single nanoparticle radius and interparticle spacing and only approximate the behavior of randomly distributed Au nanoparticles. Notably, the fraction of total absorption that occurs within the nanoparticles, and will therefore be used to drive the reaction, depends on the peak position, with a generally higher fraction observed for the lower-energy peak. Consequently, a significant enhancement is observed for the h_TiO2_ = 160 nm and h_TiO2_ = 182 nm samples, where the Au NPs exhibit absorption values of 74% and 52%, respectively.

### Characterization of Catalytic Properties of the MB Reaction

The N-demethylation reaction of MB has been previously used to investigate the mechanisms of plasmon-mediated photoreactions.^13,29^ Our previous work has shown that the yield is maximized when the LSPR overlaps with the molecular resonance (HOMO–LUMO gap) of MB, which can be identified from the absorption spectrum of MB to be approximately 530–710 nm.^13^ At resonance, energy transfer via the plasmonic near-field and hot electrons synergistically facilitate the cleavage of C–N bonds to degrade MB (Figure 5a).

**Figure 5:**
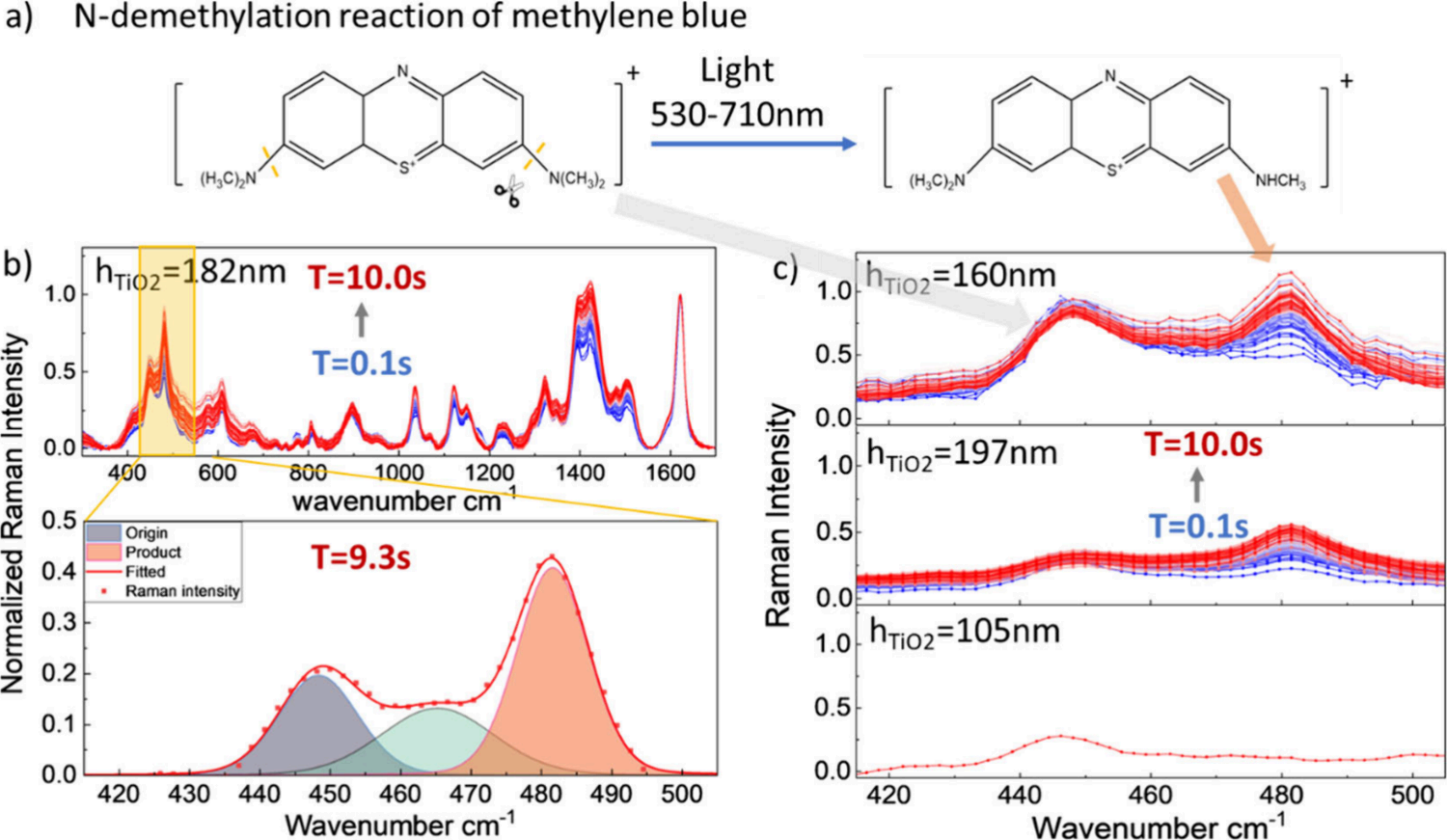
a) N-demethylation reaction of methylene blue driven via photocatalysis with light within the wavelength range of 530–710 nm. b) (top) Normalized SERS intensity of temporal measurements during a 10 s period (blue to red) on sample 5 with h_TiO2_ = 182 nm. (bottom) Fitted curves of the origin (gray area) and product (orange area) peak in the Raman spectrum at time step *t* = 9.3 s compared with measured data (red dotted line). c) Raman spectrum of h_TiO2_ = 105 nm, h_TiO2_ = 197 nm and h_TiO2_ = 160 nm samples within the wavenumber range 410–520 cm^–1^.

We monitor the dynamics of plasmon-enhanced MB N-demethylation reactions using real-time surface-enhanced Raman spectroscopy (SERS) to collect spectra at 0.1 s intervals over a 10 s period. The vibrational fingerprints reveal the temporal evolution of the chemical reactants involved in the reaction. The critical peaks used to quantify the reactants and product molecules are shown in the inset in Figure 5b. The yield can be approximated by deconvoluting and integrating these peaks as the intensity of the vibrational modes indicates the population of vibrationally excited molecules.^13^

The intensity in specific Raman peaks is related to changes in molecular concentrations of the reactants and products. As the illumination time progresses the Raman signal from products increases at the expense of the reactants, as shown in Figure 5b with the transition from blue to red curves. The excitation laser wavelength was set to 633 nm with a power of 0.68 mW, as this wavelength overlaps well with the LSPR resonance of the disordered Au NPs and the absorption spectrum of the MB solution.

At the initial time step (t = 0.1 s), the dark blue curve in Figure 5b shows the vibrational fingerprints of MB, revealing detailed chemical bond information. The peak at 448 cm^–1^ corresponds to the C–N–C skeletal deformation in MB,^29^ which undergoes cleavage during the N-demethylation photocatalytic reaction. By the final time step (t = 10.0 s), a new vibrational peak emerges at 480 cm^–1^, attributed to the skeletal deformation mode of the product thionine. The peak at 1622 cm^–1^ is associated with C–C stretching in the benzene ring^29^ which remains unchanged over the course of the reaction, allowing the spectra at different timesteps to be normalized.

To investigate the effect of tunable optical properties on the photocatalytic performance, we conducted SERS measurements on the six samples under identical illumination conditions and analyzed specific peaks related to the reaction. Figure 5c presents the temporal Raman spectrum for Au NP-cavity samples with TiO_2_ thicknesses of h_TiO2_ = 105 nm, h_TiO2_ = 160 nm, and h_TiO2_ = 197 nm.

For the h_TiO2_ = 105 nm sample, we observe negligible changes in the Raman spectra during the 10 s measurement, indicating that the reaction is significantly suppressed due to the low absorption in the Au NPs caused by the aforementioned superposition of LSPR resonance and the F–P node. In contrast, the h_TiO2_ = 160 nm sample has a measured absorption of 86% at the excitation wavelength of 633 nm (see Figure 4d). The thionine product peak intensity of this sample rose noticeably from 0.49 to 1.14, indicating a significant enhancement of the reaction due to the tuned optical properties of the Au NP-cavity. Similarly, for the h_TiO2_ = 197 nm sample, with absorption of 69% at 633 nm, the product peak intensity rose from 0.22 to 0.55 indicating that the reaction showed moderate enhancement.

To quantitatively analyze the temporal evolution of the SERS signal intensity, we estimate the product yield for the samples with varying cavity thicknesses following our previous approach.^13^ The baseline is first removed from each SERS spectrum, and peaks between 415–505 cm^–1^ are deconvoluted. The lower panel in Figure 5b shows the fitted curves for the sample with h_TiO2_ = 182 nm at the 9.3 s time step as an example. An estimate of the yield can be quantified by integrating under the peak associated with the product thionine (∼480 cm^–1^), shown as the shaded orange area in Figure 5b.

The estimated product yields as a function of time are shown in Figure 6a, illustrating the temporal evolution across the six samples. For the samples h_TiO2_ = 105 nm and h_TiO2_ = 117 nm, the yields were negligible throughout the measurement time period as the plasmonic resonance was suppressed and could not provide sufficient energy to activate the reaction. However, the other four samples with the enhanced absorption due to the hybridized modes result in yields that increase rapidly during the first 5 s and stabilize as time progresses, with variations depending on the optical properties of the individual samples.

**Figure 6:**
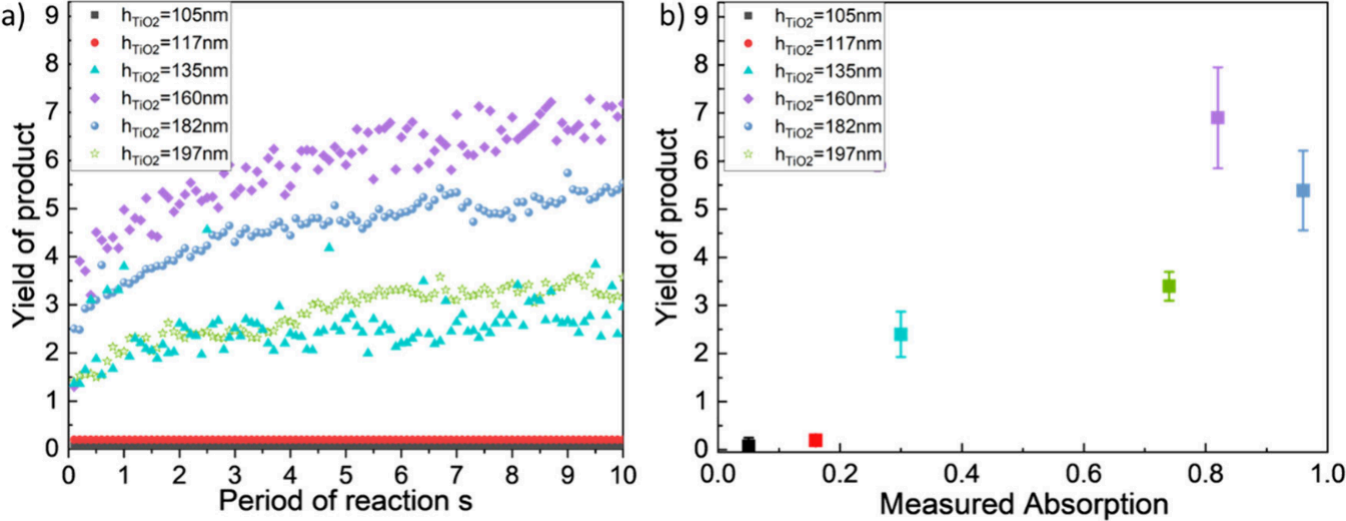
a) Yield of the six samples against the reaction processing time. b) Averaged yield at the final time step (*t* = 10 s) put in relation with the measured absorption. The error bar indicates the standard deviation for 5 measurements.

To explore the relationship between the optical and catalytic properties of the Au NP-cavities, Figure 6b plots the averaged product yield at the final time step against the measured absorption at the target wavelengths of 633 nm, with error bars representing the standard deviation from five SERS measurements taken at different points on each sample. A clear correlation is observed between estimated product yields and measured absorption. In the h_TiO2_ = 105 nm and h_TiO2_ = 117 nm samples, where absorption is below 10%, the reaction is suppressed, resulting in low product yields of 0.07 and 0.20, respectively, while absorption above 80% leads to yields of 7.18 and 5.52 for the h_TiO2_ = 160 nm and h_TiO2_ = 182 nm samples. Relative reaction yields increase by up to a factor of 102 when transitioning from samples with low optical absorption (<10%) to those with high optical absorption (80%).

We note, however, that the relationship is not monotonic: *e.g*. the maximum yield is achieved by the h_TiO2_ = 160 nm sample, despite its measured absorption being slightly lower than that of the h_TiO2_ = 182 nm sample. We attribute this to the fact that we are correlating yield against measured *total* absorption, not absorption in the Au NPs, which is the most direct measure of the hot electron transfer rate and near field strength.^13,30^ Unfortunately, it is not possible to measure this directly by using standard optical characterization techniques. Further, the absorption in the Au NPs is not expected to scale linearly with the total absorption at the target wavelength, as observed by inspection of the simulated spectra in Figure 4. In general, the fraction of the simulated total absorption that is occurring in the Au NPs is higher for the low energy peak than for the high energy peak. It is possible that a larger fraction of the measured total absorption at 633 nm in the h_TiO2_ = 160 nm TiO_2_ sample is occurring in the Au NPs, where the low energy peak aligns with the target wavelength, compared to the h_TiO2_ = 182 nm sample, where the dip between two peaks aligns with the target wavelength, leading to higher yields. A similar trend can be observed in the simulation results related to the two nonthermal effects: averaged near-field enhancement and hot electron generation rate. As shown in Figure S5a-b, the lower energy peak exhibits both a stronger averaged electric field enhancement and a significantly higher hot electron generation rate compared to the higher energy peak and the dip. Therefore, the h_TiO2_ = 160 nm TiO_2_ sample, which aligns with the lower-energy peak at 633 nm, is expected to achieve a high yield due to the enhanced performance in both nonthermal effects.

These results indicate that reaction yields can be prescriptively controlled through the modification of optical resonances. Here we emphasize that the reaction conditions for all samples are similar, employing the same catalysts, reactants, and excitation conditions. Despite this, we are able to suppress or enhance the yield by modifying the optical properties via hybridization of cavity optical modes with Au NP plasmonic resonances. In the photoreaction under study, the degradation processes are disabled, enabled, and enhanced by tuning the TiO_2_ cavity resonance wavelength against the molecular resonance (HOMO–LUMO gap) of the reactant MB.

Additionally, no complex fabrication techniques are required to shift the resonance of the hybridized mode, which was achieved by simply adjusting the duration of the TiO_2_ film deposition. By employing self-assembly to fabricate the Au NP arrays, the approach overcomes the dimension limitations imposed by top-down nanofabrication techniques and can be scaled up to the larger dimensions needed for practical devices.

Moreover, hybridized modes can exhibit spectral tunability further than demonstrated here. We have previously demonstrated through simulations that hybridized modes can be used to tailor Q factors and optimize absorption spectra for different applications.^24^ By demonstrating the prescriptive suppression and enhancement of photoreactions, our results imply that this improved flexibility in spectral engineering could be applied to more complex reactions, such as the multipathway, multibranched CO2RR.^31^

## Conclusion

We demonstrate the optical manipulation of photocatalysis by tuning the optical properties of hybridized Au NP-cavities. Near-field enhancement and hot carrier transfer induced by excitation of LSPR are used to drive a model photoreaction. By suppression or enhancement of the LSPR, catalytic behavior can be manipulated simply by modifying the optical properties of the nanostructure.

We designed Au NP-cavities that can support hybridized modes through strong coupling of the LSPR and F–P resonances. Measured absorption spectra can be varied from 4% (h_TiO2_ = 105 nm) to 92% (h_TiO2_ = 182 nm) at the target wavelength of 633 nm for the photocatalytic reaction, demonstrating control of the optical energy transfer by adjusting the thickness of the cavity layer, and hence the hybridized resonance wavelength.

The yield of the N-demethylation of MB reaction product, thionine, was monitored in real-time via SERS, and was evaluated by the area of the specific vibrational peak of product at 480 cm^–1^. This product yield increases from 0.07 for samples with suppressed absorption at the target wavelength to a maximum of 7.18 (h_TiO2_ = 160 nm) for samples with optimal spectral overlap of the hybridized mode with the MB resonance (HOMO–LUMO gap). Further, we demonstrate that reaction yields can be modulated by adjusting the absorption strength at the target wavelength, through shifts in the F–P resonance wavelength, allowing for selective enhancement or suppression of the catalytic process.

We achieve optical control of hybridized resonances using fabrication techniques with no dimensional constraints, such as self-assembled Au NPs, ensuring that this approach could be scaled for practical devices. Our findings indicate that reaction yields can be prescriptively controlled through the modification of optical resonances.

## Methods

### FEM Simulation

The far-field optical properties of the Au NP-cavity system were modeled using COMSOL Multiphysics 5.6 with the finite element method. The refractive index of TiO_2_ in this model was based on measurement of a 100 nm thin film deposited under conditions similar to those of the experimental samples, using an ellipsometer (JA Woollam M-2000D) covering wavelengths from 200 to 1700 nm. Floquet periodic boundary conditions were applied to all four surrounding surfaces to simulate a periodic array of Au NPs. The incident light was normally coupling to the nanostructure, introduced through a port with wave excitation. The total absorption was determined using the equation *1–T–R*, where *T* represents total transmittance, and *R* corresponds to total reflectance. The optical response of the nanostructure was examined as a function of the Au NP radius, interparticle gap, and TiO_2_ cavity thickness. Additional simulation results can be found in the SI. Figures S3 and S4 reveal the total absorption as a function of Au NP parameters.

### Sample Preparation

Glass slides were used as substrates for the sample preparation. Au mirror films were deposited via electron beam (E-beam) evaporation (Temescal BJD-2000) under a vacuum pressure of 1 × 10^–5^ Torr. TiO_2_ layers were then deposited onto the Au mirror by RF sputtering at an approximate deposition rate of 0.17 Å /s, with the chamber pressure maintained below 4 × 10^–6^ Torr. The thickness of the TiO_2_ cavities was regulated by adjusting the sputtering duration, as outlined in Table S1.

The Au NPs were deposited by a standard self-assembly technique. A 5 nm-thick Au film was thermally evaporated onto the top surface of the TiO_2_ cavity (Kurt Lesker NANO 36 system) at a deposition rate of 1 Å/s, while keeping the chamber pressure below 1 × 10^–5^ Torr. The samples were then annealed at 500 °C for 2 h, causing the Au film to reshape into segregated nanoislands. This morphological transformation occurred exclusively on the top of the Au layer. More information on this can be found in Figure S6.

To facilitate molecular attachment, the samples were immersed overnight in a 5 ppm methylene blue solution, leading to a random distribution and orientation of the molecules on the Au NPs.^13^

### Microspectrophotometer Measurement

A bespoke microspectrophotometer was used to characterize the far-field optical properties of the Au NP-cavities. A Xenon Arc Light Source (Thorlabs SLS401) provided illumination across the full visible spectrum, covering wavelengths from 300 to 800 nm. A 10× objective lens (Olympus Mplan N) focused the light onto the sample and collected the reflected and transmitted light within an acceptance angle of 22°, which was then directed to a fiber spectrometer (StellarNet Inc. BLACK-Comet-SR). To minimize random noise, the spectrum was integrated over 100 s and averaged over five measurements.

## Supplementary Material



## Notes

We present the supplementary simulation results for individual Au nanoparticles and the TiO_2_ thin film in Figure S1 to investigate individual resonances prior to mode hybridization. A detailed simulation approach is provided, including key parameters such as the refractive index of TiO_2_ in Figure S2. The absorption of the coupled Au NP-cavity is investigated with various Au NP parameters and cavity thickness, which are present in Figures S3 and S4. And the near-field enhancement and hot-electron generation rate are calculated in Figure S5. Additionally, sample preparation details are outlined, with the process illustrated in Figure S6. Table S1 summarizes the relationship between sputtering processing time and cavity thickness. The morphology of the Au nanoparticles is discussed in their radius and interparticle gaps, as shown in Figures S7 and S8. And the strong coupling strength determined with measured spectrum in Table S2. Moreover, the optical performance of Au mirror film is investigated both before and after annealing, which are exhibited in Figure S9. Some supplementary SERS measurements are shown in Figures S10–S12 (PDF)

## Abbreviations

LSPR: localized surface plasmon resonance
HOMO: highest occupied molecular orbital
LUMO: lowest unoccupied molecular orbital
CO2RR: carbon dioxide reduction reaction
Au-NP cavity: Au nanoparticle-cavity
F–P resonance: Fabry–Pérot resonances
MB: methylene blue
FEM: finite element method
SEM: scanning electron microscope
SERS: surface-enhanced Raman spectroscopy.
