# Supplementary material for: Coupled Au Nanoparticle-Cavity Nanostructures for Precise Control in Resonance-Driven Photocatalytic Reactions
Source: ACS Nano. 2025 Jul 11;19(28):25821–9. doi: 10.1021/acsnano.5c04020 (PMC12291579; doi:10.1021/acsnano.5c04020)
Supplement: Supplementary file 1 [file nn5c04020_si_001.pdf]

Supporting information

Coupled Au nanoparticle-cavity nanostructures for precise control in resonance-driven photocatalytic reactions

Ning Lyu [a b], Anjalie Edirisooriya [b], Zelio Fusco [b], Shenyou Zhao [c], Fiona J. Beck [b], Christin David [a d*]*

a. Institute of Solid State Theory and Optics, Friedrich-Schiller-Universität Jena, 07743 Jena, Germany

b. School of Engineering, Australian National University, ACT 2601, Australia

c. School of Electronic Engineering, Xi'an University of Posts and Telecommunications, Xi'an 710121, China

d. University of Applied Sciences Landshut, Am Lurzenhof 1, 84036 Landshut, Germany

* Corresponding authors: christin.david@uni-jena.de (C. David); fiona.beck@anu.edu.au (F.J. Beck)

Details on performed optical simulations

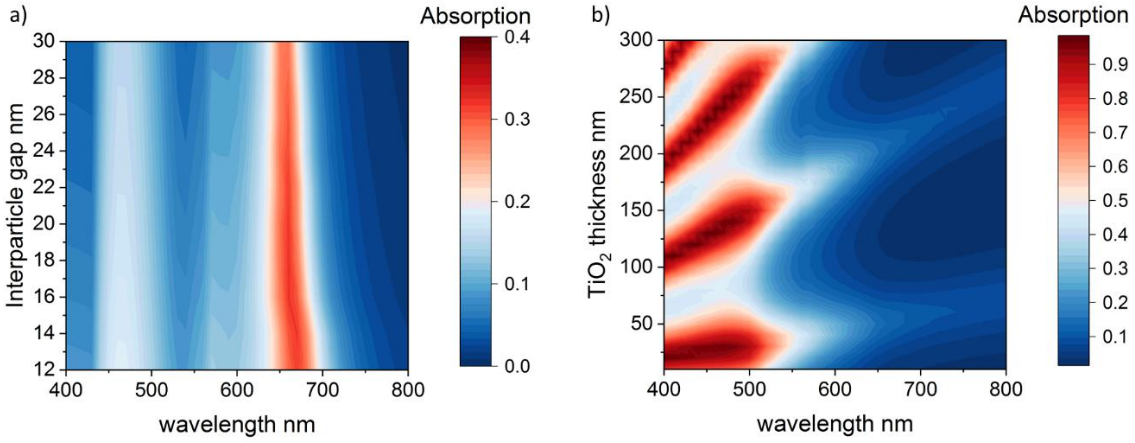

Figure S1 Simulated absorption of localized surface plasmon resonance (LSPR) and Fabry–Pérot (F-P) resonance. a) Total absorption of Au nanoparticles on a TiO_2 layer of infinite thickness, with interparticle gaps increasing from 12 to 30 nm. b) Total absorption of a TiO_2 cavity on an Au mirror layer, with varying cavity thickness from 10 to 300 nm.

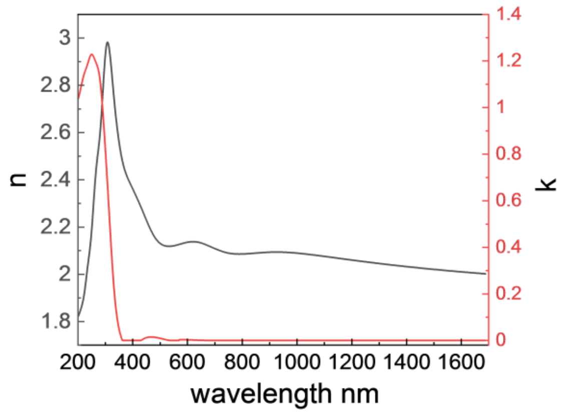

Figure S2 Refractive index of TiO_2 thin film measured with ellipsometer showing its real part n (black curve) and imaginary part k (red curve).

The optical properties of the studied Au nanoparticle-cavity (Au NP-cavity) designs were simulated using COMSOL Multiphysics 5.6 with the finite element method (FEM). We calculated the absorption spectra for Au nanoparticles (Au NPs) as a function of the interparticle gap and the TiO_2 nanocavity layer as a function of its thickness shown in Figure

S1a) and S1b), respectively, and the Au nanoparticle-nanocavity system as a function of the TiO_2 layer thickness (Figure 1b in the main article), examining the localized surface plasmon resonance (LSPR), Fabry-Pérot (F-P) resonance, and the hybridized resonance resulting from strong coupling between these two modes.

The refractive index of TiO_2 was measured with a JA Woollam M-2000D ellipsometer from 200-1700 nm on a 100 nm thickness thin film, as shown in Figure S2. These data were applied in the material model of the optical simulations, which also demonstrates that the phase of TiO_2 is anatase.

For the simulation of Au NPs in Figure S1a), the model consisted of a 14 nm in radius for Au NP on an infinite TiO_2 layer, an average size based on the characterization results detailed below. Floquet periodic boundary conditions were applied to the four surrounding surfaces, creating a periodic array of Au NPs. The nanoparticle gap varied from 12 to 30 nm which aligns well with the measured distribution. The LSPR peak exhibited a slight blue shift as the interparticle gap increased and then stabilized at around 655 nm. For the sample with an average interparticle gap of 22 nm, the LSPR peak occurred at 659 nm.

For the nanocavity model in Figure S1b), a TiO_2 thin layer was placed on a 200 nm Au mirror, which had thickness varying from 10 to 300 nm. The TiO_2 nanocavity supports constructive F-P resonances at specific wavelengths which red-shifted as the cavity thickness increased. The bandgap of TiO_2 and interband electronic transitions of Au mirror film[1] limits the absorption wavelength of the cavity, causing a cut-off in absorption around 550 nm.

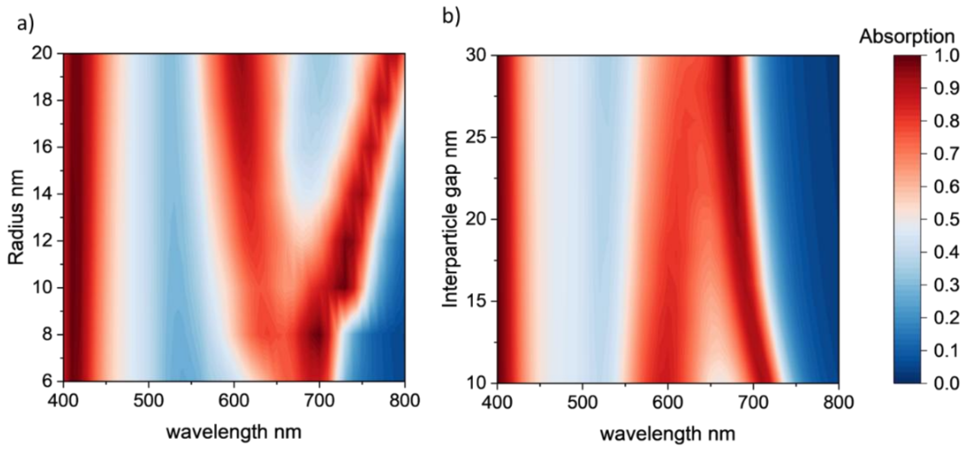

Figure S3 Simulated absorption of Au NP-cavities with varying NP parameters: (a) Total absorption of Au NP-cavities as a function of NP radius, with h_{TiO2} = 200 nm and an interparticle gap of 8 nm; (b) Total absorption of Au NP-cavities as a function of interparticle gap, with h_{TiO2} = 200 nm and r = 14 nm.

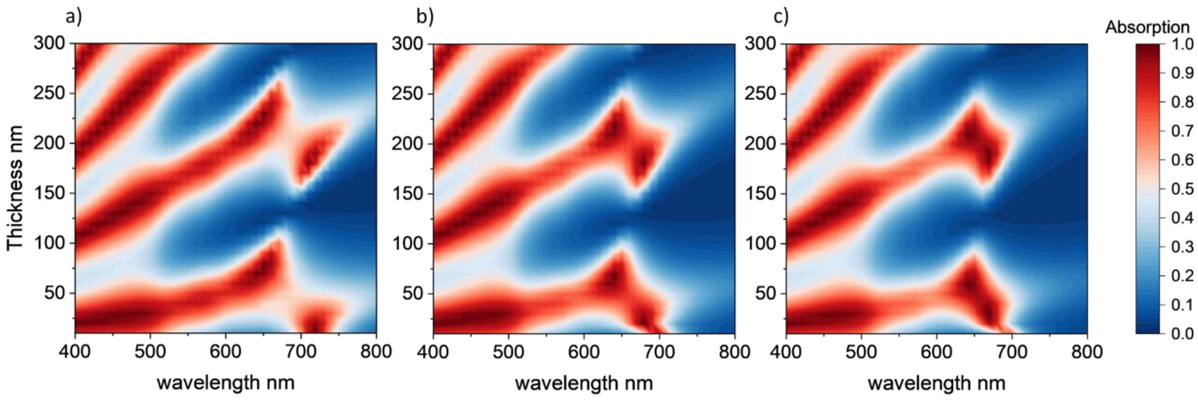

Figure S4 Simulated total absorption of Au NP-cavities with various interparticle gaps. The thickness of TiO_2 cavities varying from 10 to 300 nm a) Au NPs with r = 14 nm and gap = 8 nm; b) Au NPs with r = 14 nm and gap = 21 nm; c) Au NPs with r = 14 nm and gap = 30 nm.

Moreover, as shown in Figure S3, the total absorption of the Au NP-cavity system is calculated as a function of NP parameters, specifically the NP radius and interparticle gap. The coupling strength, $h\Omega$, is defined as the energy difference between the high- and low-energy modes under strong coupling conditions and is determined by both the NP radius and the interparticle gap. In Figure S3a), the coupling strength increases with enlarging of NP radius.

Conversely, as the interparticle gap increases, the density of dipole moments decreases, resulting in a reduction in the splitting energy, as illustrated in Figure S3b). Additionally, this variation in coupling strength is illustrated in the absorption spectrum for designs with varying TiO_2 thicknesses, ranging from 10 to 300 nm, as presented in Figure S4. A similar trend is observed in mode splitting energy, which decreases with increasing interparticle gaps. A comprehensive investigation of the strong coupling associated with NP parameters has been study in the previous work of our group.[2]

Several simulations are performed to estimate the photocatalytic reactivity among samples, which focus on two non-thermal effects of LSPR: near-field enhancement and hot electron transfer. Specifically, the averaged squared electric field enhancement is evaluated:[3]

$$\left(\frac{E}{E_0}\right)^2 = \frac{1}{a^2\left|\vec{E}_0\right|^2}\int_0^a dx \int_0^a dy\left|\vec{E}(x,y,z)\right|^2$$

where E_0 is the reference electrical field and a^2 is the area of calculation surface. In the Au NP–cavity model, the electric field enhancement is evaluated on the surface of the Au NP, as illustrated in Figure S5a). As expected, the enhancement is concentrated near the plasmonic resonance wavelength. The coupling between the LSPR and F–P cavity modes modulates the electric field enhancement as the cavity thickness increases, resulting in the a dual-peak.

Additionally, the rate of hot electron generation is determined by the component of the electric field normal to the nanoparticle surface:[2]

$$\text{Rate}_{\text{high-energy}} \approx \frac{2}{\pi^2} \times \frac{q^2 E_f^2}{\hbar}\frac{1}{(\hbar\omega)^3}\int_S |E_{\text{normal}}(x,y,z)|^2 ds$$

where q, E_f and $\hbar$ are the charge of an electron, Fermi energy of gold and reduced Planck's constant, respectively. The illumination intensity used in the simulation is based on the laser intensity applied in the SERS measurements. As shown in Figure S5b), the hot electron generation rate can also be modulated by varying the cavity thickness, owing to the strong coupling effect present in the samples. A significantly higher generation rate is observed at the

lower-energy resonance frequency, which explains the peak reaction yield observed in the sample where the target wavelength aligns with this lower-energy peak.

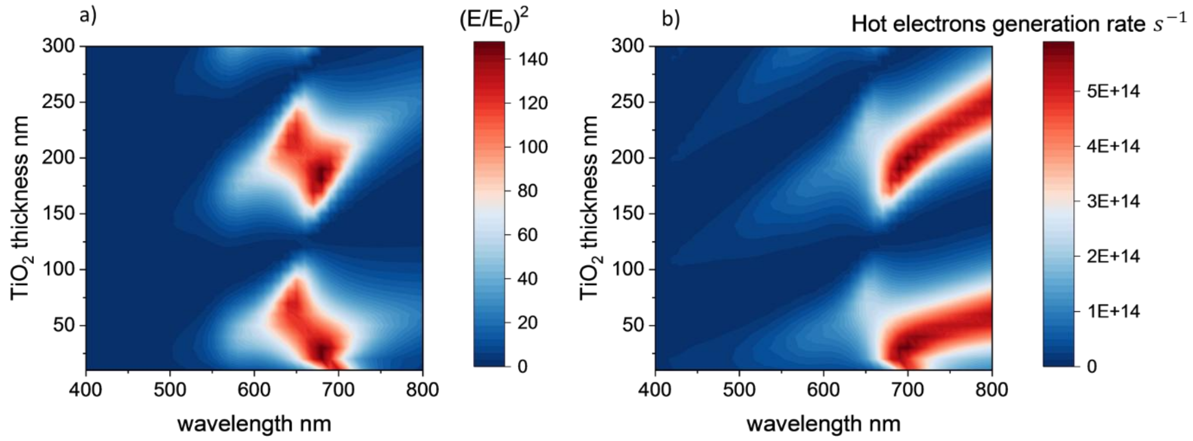

Figure S5 a) Averaged squared electric field enhancement of the Au NP–cavity structure as a function of cavity thickness. The enhancement is concentrated around the plasmonic resonance wavelength, and strong coupling with the F–P resonance leads to an energy splitting. b) Hot electron generation rate in the Au NP–cavity structure as a function of cavity thickness, simulated under the illumination intensity used in the SERS measurements. A significantly higher generation rate is observed at the lower-energy resonance frequency.

Sample preparation

The substrates used for the samples were glass slides as shown in Figure S6a). Au mirror films were prepared using a Temescal BJD-2000 electron beam (E-beam) evaporator with a film thickness of 200 nm in Figure S6b). TiO_2 nanocavities were deposited onto the Au mirror using RF sputtering (in Figure S6c)). The thickness of the TiO_2 cavities (h_{TiO2}) was controlled by the sputtering duration, as shown in Table S1.

Figure S6d) – e) reveal the self-assembly approach of Au nanoparticles. A 5 nm Au thin film was deposited onto the top surface of the TiO_2 cavity using a Kurt Lesker NANO 36 thermal evaporator at a deposition rate of 1 Å/s. The samples were then annealed at 500°C for 2 hours to form segregated Au nanoislands. During this step, only the Au thin film on the top surface

underwent morphological changes. Finally, the samples were immersed overnight in a 5 ppm methylene blue solution to attach the molecules to the Au NPs resulting in a random distribution and orientation of molecules as shown in Figure S6f).[4]

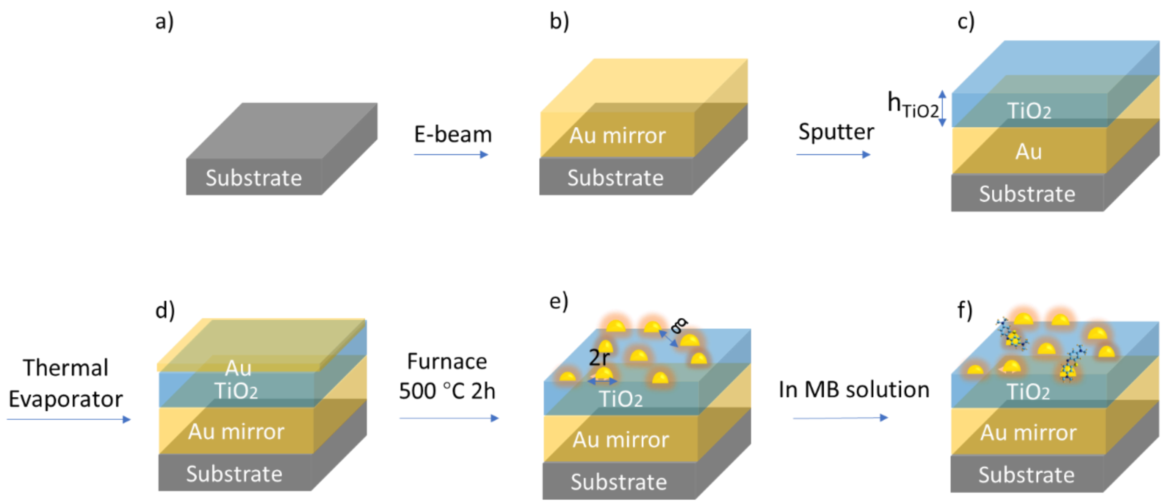

Figure S6 Fabrication process of Au nanoparticle-nanocavity designs. The optical performance of the different designs is mainly determined by the thickness of the TiO_2 cavity (h_{TiO2}), Au NP size (r) and interparticle gap (g) of Au NPs.

Table S1 Sputter processing time and TiO_2 cavity thickness

NO.	Processing time of sputtering	Averaged TiO_2 thickness nm
**1**	2 h	105.03 ± 1.33
**2**	2 h 10 min	117.49 ± 2.34
**3**	2 h 20 min	135.49 ± 1.68
**4**	2 h 40 min	159.94 ± 1.43
**5**	3 h	181.81 ± 2.31
**6**	3 h 20 min	197.56 ± 1.51

The thickness of the TiO_2 cavities was measured using a JA Woollam M-2000D ellipsometer on reference samples deposited on glass slides under the same sputtering duration.

Measurements were conducted across a wavelength range of 200–1700 nm. The reflective spectrum was collected for both s- and p-polarized light at three incident angles: 55°, 65°, and 75°. The fitting of the measured amplitude ratio and phase shift of the complex reflectance ratio was initiated using a model based on the measured refractive index of TiO_2, as shown in Figure S2. The Table S1 presents the average values from three measurements taken at different positions on the samples. The thickness of the TiO_2 cavities increased uniformly with the extended sputtering duration, exhibiting an average deposition rate of approximately 0.17 Å/s.

Morphology of the Au NP layers

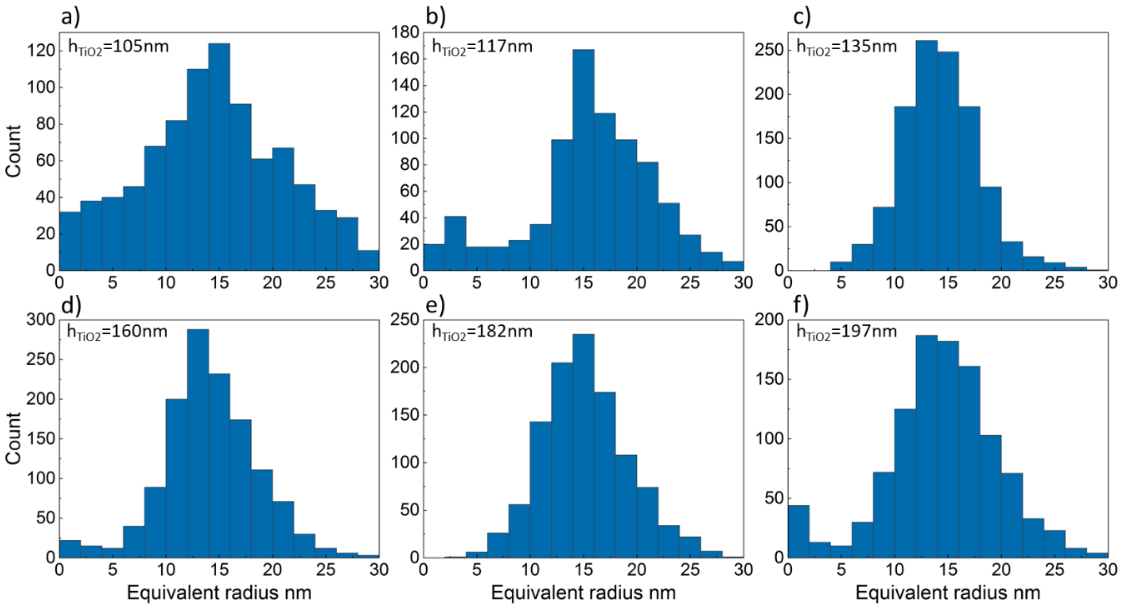

Figure S7 Histograms of equivalent radius for six samples evaluated using corresponding SEM images. The average radii are 11.0 (h_{TiO2} = 105 nm), 11.7 (h_{TiO2} = 117 nm), 13.3 (h_{TiO2} = 135 nm), 14.1 (h_{TiO2} = 160 nm), 15.2 (h_{TiO2} = 182 nm), 14.4 (h_{TiO2} = 197 nm), respectively.

As demonstrated in Figure S3a) and in our previous study, the parameters of Au NPs determine their optical properties.[2] We analyzed the morphology of Au NPs using scanning electron microscopy (SEM) images from six samples. The equivalent radius of randomly structured Au NPs was calculated based on the radius of a circle with the same surface area. The areas of the Au NPs were determined using the 'Analyze Particles' function in ImageJ.

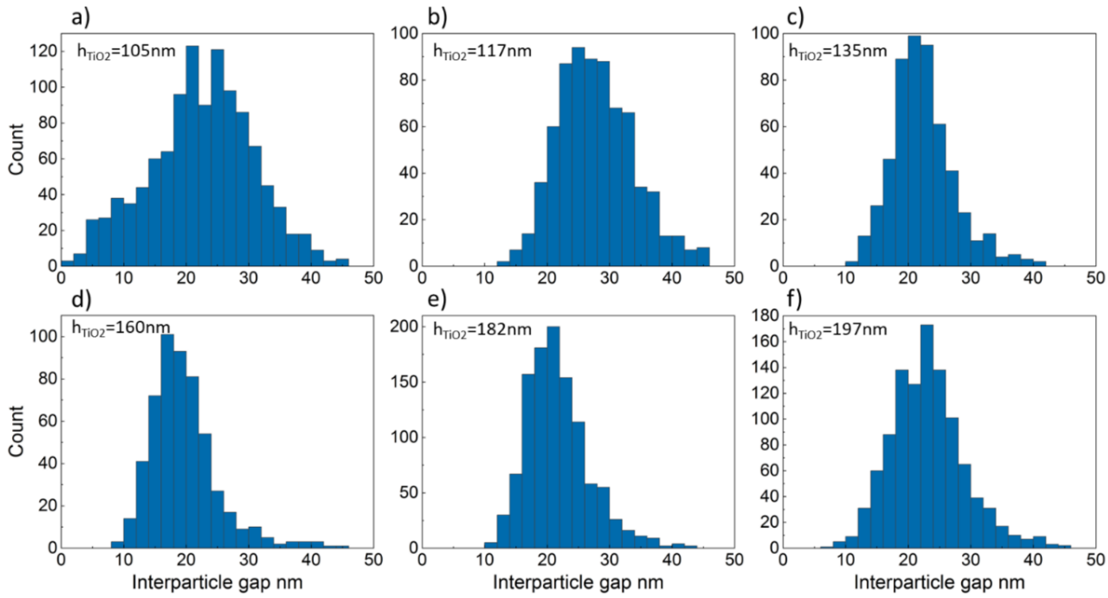

Figure S8 Histograms of interparticle gap for six samples, evaluated based on the five nearest adjacent nanoparticles from related SEM images. The average gaps are 24.4 (h_{TiO2} = 105 nm), 24.4 (h_{TiO2} = 117 nm), 21.9 (h_{TiO2} = 135 nm), 19.7 (h_{TiO2} = 160 nm), 21.7 (h_{TiO2} = 182 nm), 23.4 (h_{TiO2} = 197 nm), respectively.

Figure S7a) - f) display histograms of the nanoparticle radius across the six samples. The equivalent radius is normally distributed, ranging from 1 to 30 nm, with an average value of approximately 14 nm. Additionally, interparticle gaps were calculated using the following equation:

$$g_{interparticle} = d_{interparticle} - r_1 - r_2$$

where $d_{interparticle}$ is the center-to-center distance between two neighboring nanoparticles, and r_1 and r_2 are the equivalent radius of these two nanoparticles. Figure S8a) - f) show histograms of interparticle gaps of five closest neighbors across the six samples. The interparticle gaps range from 10 to 50 nm with an average value of approximately 22 nm.

Strong coupling strength evaluation

The coupling strength, collected for selected samples in Table S2, reaches its maximum value of 79 GHz for the sample with h_{TiO2} = 160 nm. At this thickness, the plasmonic resonance

exhibits both strong spatial and spectral overlap with the cavity mode, enabling the most efficient coupling from samples produced in this study. As the TiO_2 thickness increases, the cavity resonance undergoes a redshift, leading to spectral misalignment with the plasmonic resonance. The coupling strength decreases. For the sample with h_{TiO2} = 197 nm, the coupling strength is reduced to 51 GHz.

Table S2 Resonance frequency and coupling strength of strong coupling samples

h_{TiO2} nm	Upper resonance frequency ω_+ GHz	Lower resonance frequency ω_- GHz	Coupling strength Ω GHz
160	551	472	79
182	512	455	57
197	486	435	51

Au mirror layer during annealing

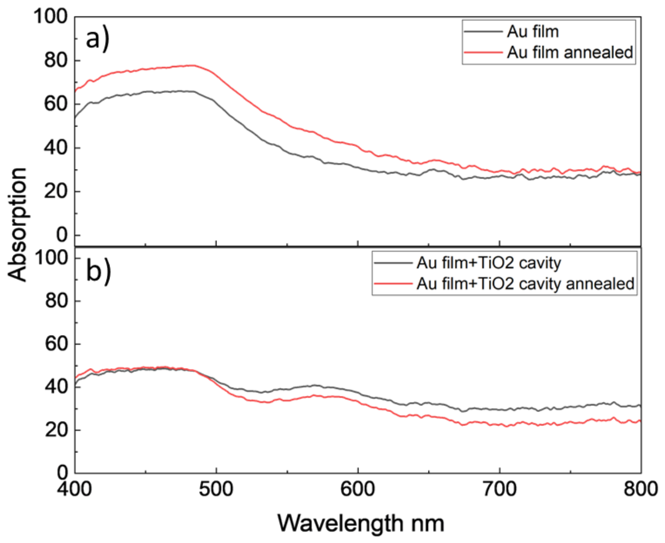

Figure S9 a) Absorption of the Au mirror layer before and after annealing 500°C for 2 hours b) Absorption of the Au mirror layer with 200nm TiO_2 cavities before and after annealing 500°C for 2 hours.

The stability of the Au mirror layer was investigated using two samples: a Au film and an Au film deposited with a 200 nm TiO_2 cavity layer. The total absorption of each sample was measured before and after annealing, as presented in Figure S9. The annealing conditions were

consistent with the values in the self-assembly process of Au NPs, conducted at 500°C for 2 hours. The absorption difference between the two samples was found to be less than 8%, which is considered negligible. Therefore, during the fabrication of the Au NP-cavity structure (in Figure S7e)), morphological transformations of the Au NPs are expected to occur only within the top 5 nm of the Au film.

Supplementary experiments for SERS measurement

The Raman spectra of the h_{TiO2} = 160 nm Au NP-cavity are measured under varying laser powers, ranging from 0.07 mW to 0.68 mW. The product yield is represented by the intensity of the vibrational peak at 480 cm^{-1} measured over a 30-second period. As the laser power increases, the plasmonic resonance of the Au NPs is enhanced, resulting in a greater intensity change at the product peak, which reflects an increase in product yield.

Also, the Raman spectrum was measured for MB adsorbed on an h_{TiO2} = 170 nm TiO_2 film deposited on the Au mirror substrate. In the absence of plasmonic resonance, the Raman signal intensity was significantly reduced. The black curve in Figure S10b) shows the signal magnified 10 times. A higher laser power (13.6 mW) and a longer integration time (20 s) are employed in this measurement to obtain a detectable signal. This spectrum is compared with that from the Au NP-cavity sample with the same TiO_2 thickness; the green curve shows the spectrum under 0.68mW 633 nm laser after 20 seconds (with a 1 s integration per spectrum). Specific Raman peaks can be identified, such as the C–C stretching in the benzene ring at 1622 cm^{-1} and the C–N–C skeletal vibration in MB at 448 cm^{-1}. Notably, the product peak at 480 cm^{-1} is significantly diminished in the spectrum without Au NPs. These results demonstrate that the plasmonic resonance of Au nanoparticles plays a key role in creating the cavity by trapping light and enhancing the photocatalytic degradation of MB taking place at the setup's interface.

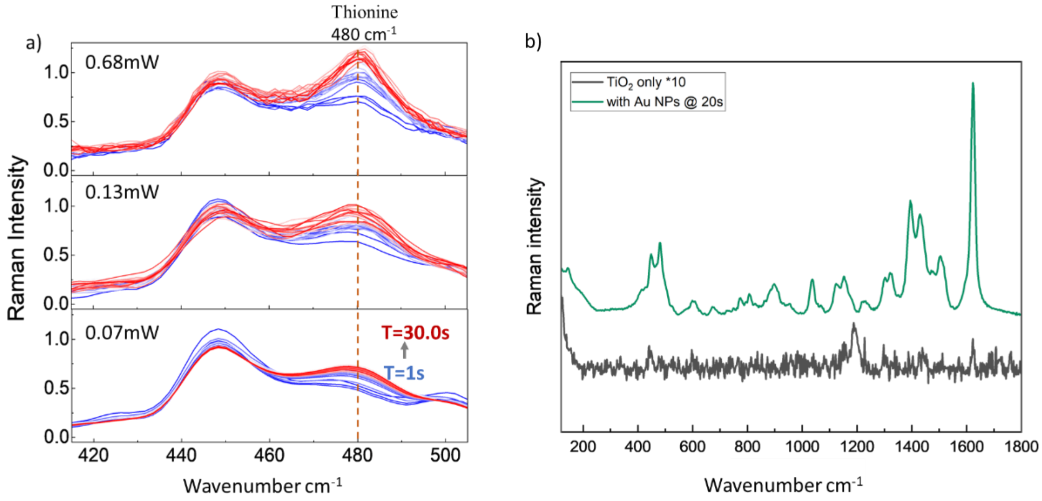

Figure S10 a) Normalized Raman spectrum of the h_{TiO2} = 160 nm Au NP-cavity under various laser powers shows the intensity changes of a product-specific peak at 480 cm^{-1}, with spectra shifting from t = 1 s (blue curves) to t = 30 s (red curves). The peak intensity increases with laser power, indicating an enhancement in reaction rate or yield due to photocatalysis. b) For comparison, the Raman spectrum of MB adsorbed on a h_{TiO2} = 170 nm TiO_2 film without Au NPs was measured using a 13.6 mW, 633 nm laser with a 20 s integration time. The MB Raman signal is very weak, comparable to the measurement noise level. The black curve shows the signal from the Au NP-cavity sample under 0.68mW 633 nm laser at the 20th s (green curve).

The laser wavelength for photocatalysis in this study is determined by the properties of the reactant, MB, as well as the available laser source for Raman spectroscopy. The absorption and emission spectra of MB in solution can be found from our previous work.[5] The absorption range, approximately 530–710 nm, is attributed to π-electron resonance of the sulphur atoms and $\pi - \pi^*$ transitions of the benzene ring in monomeric MB. In selecting the laser source for Raman spectroscopy, we considered wavelengths of 532 nm, 633 nm, and 785 nm. Among these, only the 633 nm laser shows strong overlap with the MB absorption spectrum, enabling LSPR in the Au NP-cavity design to drive the reaction. In contrast, MB exhibits low absorption at 532 nm and 785 nm. Therefore, in Figure S11, the Raman signal strength varies significantly with the excitation wavelength, with the strongest signals observed at 633 nm. When the system

is excited with a higher-energy photon at 532 nm, the vibrational fingerprints of MB are still visible, but the product peak shift is negligible. Similarly, under excitation with lower-energy photons at 785 nm, the overall Raman signal is significantly weaker, and no noticeable peak shift is observed.

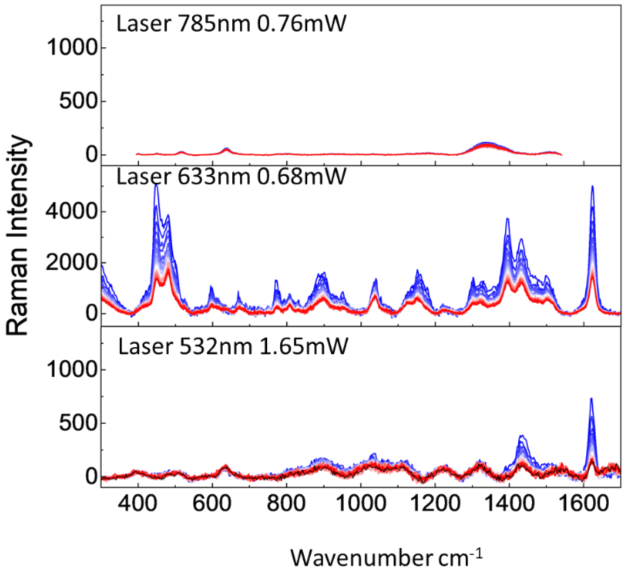

Figure S11 Raman spectrum of the h_{TiO2} = 160 nm Au NP-cavity under different laser wavelengths reveals varying reactivity over a 30-second measurement period. The reaction rate is significantly enhanced under 633 nm excitation, which correlates with the absorption spectrum of MB.

To investigate the long-term stability of the samples, we redid the SERS measurement for the same six samples discussed in this article, as shown in Figure S12. The samples were originally prepared 13 months ago. To reintroduce the probe molecules, the samples were immersed overnight in a 5 ppm MB solution, allowing MB molecules to adsorb onto the surface of the Au nanoparticles. After this long period, the residual MB was completely degraded. The yields were calculated based on the specific peak of thionine at 480 cm^{-1}. After a 30s measured period, a similar phenomenon is observed among six samples as in the original experiments. The peak yield provided by the sample h_{TiO2} = 160 nm reaches 12.9 at the final timestep (t =

30 s). The sample h_{TiO2} = 182 nm has the second highest yield at 7.0. In contrast, the h_{TiO2} = 105 nm and h_{TiO2} = 117 nm have an extremely low yield and the change is negligible, as the reaction is disabled in these samples. These results demonstrate that the samples retained similar photocatalytic functionality after being stored at room temperature for 13 months.

However, some additional limitation is applied to this measurement as these samples were immersed in MB solution twice. The residuals provide an additional error to the SERS measurement, i.e. the thionine molecules attached to the Au NPs surface, which have not been completed removed. The concentration of MB molecules is difficult to estimate and compare for these samples.

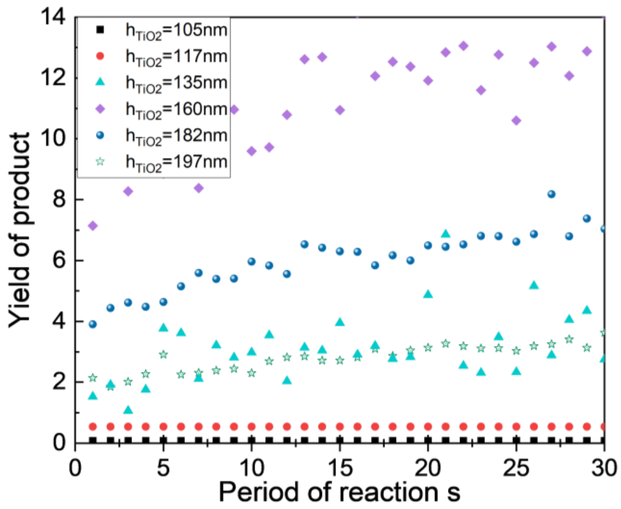

Figure S12 Reaction yields of the six samples as a function of processing time. The samples were prepared 13 months ago, and the results are based on a second Raman measurement after reintroducing MB molecules in a separate preparation step.

REFERENCES

(1) Derkachova, A.; Kolwas, K.; Demchenko, I. Dielectric Function for Gold in Plasmonics Applications: Size Dependence of Plasmon Resonance Frequencies and Damping Rates for Nanospheres. *Plasmonics* **2016**, *11* (3), 941–951.

(2) Zhao, S.; Fusco, Z.; Beck, F. J. Strong and Tunable Absorption in Coupled Nanoparticle–Cavity Systems for Plasmonically Enhanced Hot Electron Devices. *Optica* **2022**, *9* (9), 1084.

(3) David, C. TiO2 Self-Assembled, Thin-Walled Nanotube Arrays for Photonic Applications. *Materials* **2019**, *12* (8), 1332.

(4) Doan, M. Q.; Anh, N. H.; Quang, N. X.; Dinh, N. X.; Tri, D. Q.; Huy, T. Q.; Le, A.-T. Ultrasensitive Detection of Methylene Blue Using an Electrochemically Synthesized SERS Sensor Based on Gold and Silver Nanoparticles: Roles of Composition and Purity on Sensing Performance and Reliability. *J. Electron. Mater.* **2022**, *51* (1), 150–162.

(5) Fusco, Z.; Catchpole, K.; Beck, F. J. Investigation of the Mechanisms of Plasmon-Mediated Photocatalysis: Synergistic Contribution of near-Field and Charge Transfer Effects. *J. Mater. Chem. C* **2022**, *10* (19), 7511–7524.
